# Genetic mouse models to study blood–brain barrier development and function

**DOI:** 10.1186/2045-8118-10-3

**Published:** 2013-01-10

**Authors:** Fabien Sohet, Richard Daneman

**Affiliations:** 1 UCSF Department of Anatomy, 513 Parnassus Ave HSW1301, San Francisco, California, 94143, USA

**Keywords:** Blood–brain barrier, Mouse models, Endothelial cells, Neuro-vascular unit, Astrocytes, Pericytes, Central Nervous system

## Abstract

The blood–brain barrier (BBB) is a complex physiological structure formed by the blood vessels of the central nervous system (CNS) that tightly regulates the movement of substances between the blood and the neural tissue. Recently, the generation and analysis of different genetic mouse models has allowed for greater understanding of BBB development, how the barrier is regulated during health, and its response to disease. Here we discuss: 1) Genetic mouse models that have been used to study the BBB, 2) Available mouse genetic tools that can aid in the study of the BBB, and 3) Potential tools that if generated could greatly aid in our understanding of the BBB.

## Review

### Introduction

The blood–brain barrier (BBB) is a functional physiological structure formed by the blood vessels of the central nervous system (CNS) that tightly regulates the exchange of molecules, ions and cells between the blood and the CNS, and is critical for maintenance of homeostasis within the nervous tissue. Many of the properties of the BBB are possessed by the endothelial cells (ECs) that form the walls of the blood vessels, and these properties are tightly regulated by both neural and immune cells. Important BBB properties include: 1) CNS ECs are joined together by tight junctions (TJs) which create a paracellular barrier, 2) CNS ECs undergo extremely low rates of transcytosis creating a transcellular barrier to hydrophilic molecules, 3) CNS ECs express transporters to efflux potential toxins from the CNS, 4) CNS ECs express selective transporters to deliver specific nutrients to the CNS, 5) CNS ECs express very low levels of leukocyte adhesion molecules limiting the entry of immune cells into the CNS. ECs interact with immune cells in the blood, as well as different cells within the CNS parenchyma, including pericytes, astrocytes, macrophages, microglia and neurons, and these interactions are important to regulate the formation of the BBB during development, the function of the BBB during health, and the response of the BBB to injury and disease.

In this review we will discuss mouse genetic models that can be utilized to study the BBB during health and disease. First we will discuss selected genetic models that have been used to identify novel aspects of BBB function including endothelial barrier function, CNS angiogenesis and BBB development, and interactions of different cell types within the neuro-vascular unit (see Additional file 1: Supplementary Table 1. Genetic mouse models to study the BBB). In the second section we will discuss current genetic tools available for analysis of BBB function. In the final section we will suggest several potential genetic tools that if generated could greatly increase our ability to study and understand the BBB.

### Types of genetic mouse models

**Figure 1:**
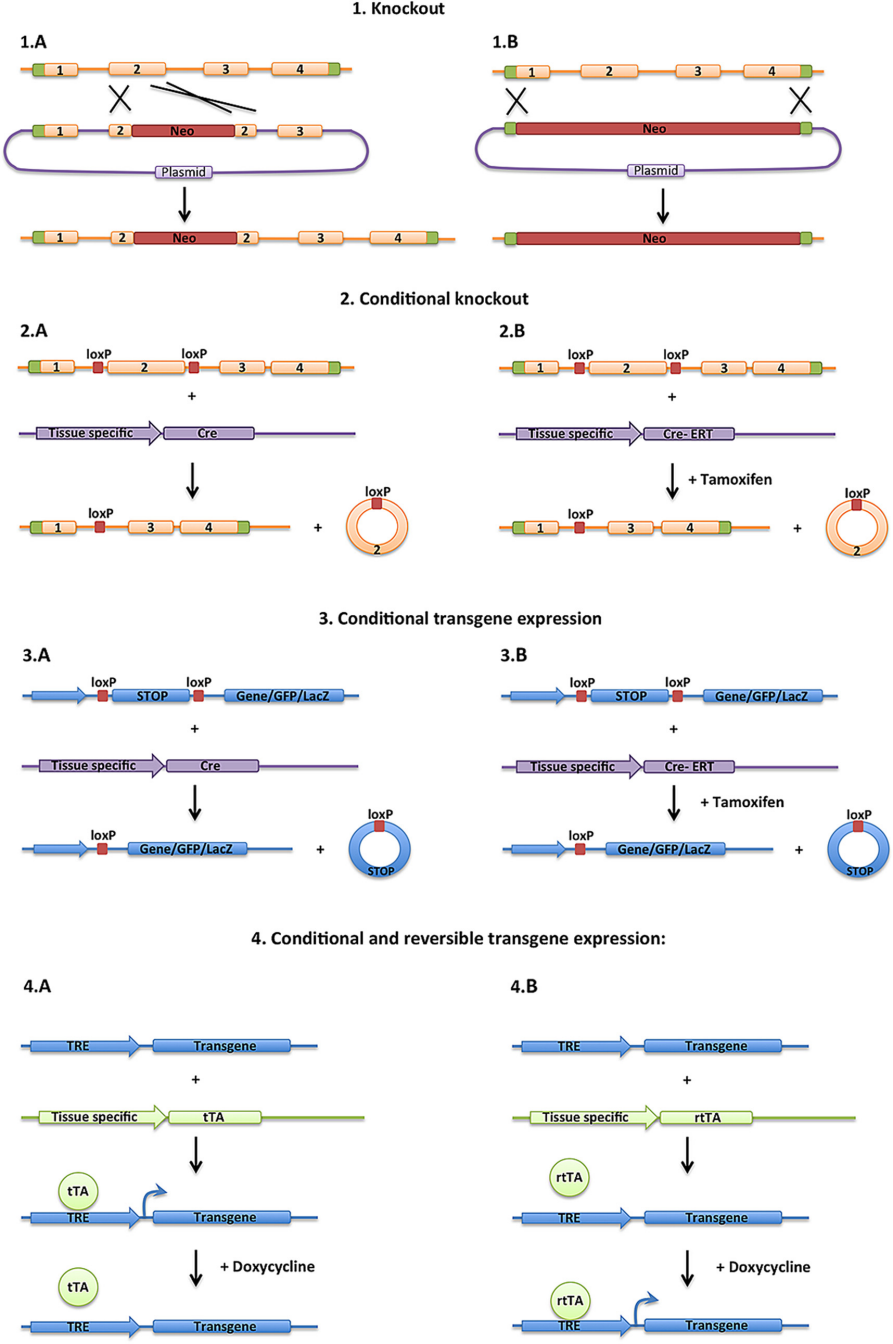
**Representation of genetic mouse models.** 1) Knockout out of specific genes. A neomycin cassette is inserted by homologous recombination either into an exon (**1A**) of a gene of interest or replacing the whole gene of interest (**1B**). 2) Methods to control the cell specificity and timing of gene deletion. Homologous recombination is used to insert lox-p sites surrounding an exon of the gene of interest. To conditionally delete the gene in a specific cell type, the Cre recombinase is expressed by a tissue specific promoter and deletes the loxP flanked region (**2A**). To regulate the timing, one can use a transgene encoding a Cre recombinase fused with the modified estrogen receptor (Cre-ERT) that will move into the nucleus upon injection of tamoxifen (**2B**). 3) Ectopic expression of a transgene. A common method is to utilize a lox-stop-lox cassette which can be removed by Cre recombinase. A transgene is generated with a stop codon that is flanked with two loxP sites upstream of the transgene of interest. The transgene can be introduced to the genome at a specific locus by homologous recombination or randomly inserted in the genome. When the Cre recombinase deletes the stop codon, the transgene can be transcribed (**3A**). This technique is available with the Cre-ERT system (**3B**). 4) To reversibly express a transgene. A common method is the use of the TRE/tTA or TRE/rtTA systems. A transgene is generated with the gene of interest downstream of the tetracycline responsive element (TRE). A second transgene is generated with a tissue specific promoter controlling the expression of tTA (Tet-Off, **4A**) or rtTA (Tet-ON, **4B**). For Tet-OFF, the tTA activates transcription of the transgene downstream of the TRE promoter, only in the absence of doxycycline. For Tet-ON, the rtTA activates transcription of the transgene downstream of the TRE promoter only in the presence of doxycycline.

In general, mouse genetic models fall under two categories: gene silencing or ectopic gene expression (Figure 1). Published mouse lines can be found in the Mouse Genomic Informatics (MGI) data base (http://www.informatics.jax.org/).

For gene silencing, the most common tools include mouse knockout or conditional knockout technologies. Mouse knockout models use homologous recombination to delete a specific gene, or a section of a specific gene, from its endogenous chromosomal locus. This technique leads to mice that lack the specific gene in all cells, and has widely been utilized to study the role of genes in mouse development and function. For genes on the somatic chromosomes, each mouse receives a chromosome from each parent, and thus one can create homozygous knockout mice in which alleles on both parental chromosomes are mutated or heterozygous mice in which only one allele is mutated. For genes on the sex chromosomes the details are more complicated. For instance, males only receive one X-chromosome from their mother, and thus for genes on the X-chromosome males can be either mutant or wild type, but not heterozygous. Females receive an X chromosome from each parent, and thus can be wild type, heterozygous or homozygous for mutant alleles, however because of X inactivation, heterozygous mutation can lead to mosaicism as a different X chromosome may be inactivated in different cells.

More recently, generation of conditional mutant mouse lines has allowed for spatial and temporal control over gene silencing. Specifically, homologous recombination is used to flank a critical exon (or exons) within a specific gene with lox-p sites. The lox-p sites do not alter gene function, but upon expression of a Cre recombinase gene, the recombinase deletes the section of the gene flanked by the lox-p sites. Therefore, expression of Cre-recombinase by transgenics, viral infection or other methods can control the cell specificity of the gene deletion. Further control of the timing of gene deletion can be achieved by using a CreERT or CreERT2 recombinase, in which the recombinase is fused to a modified estrogen receptor and thus is only targeted to the nucleus upon injection of tamoxifen [1]. Therefore use of the CreERT allows for spatial control (where the CreERT is expressed) and temporal control (when tamoxifen is injected) of gene deletion. The Cre/lox systems irreversibly delete sequences flanked by lox-p sites, and thus several different methodologies have been used to deliver double-stranded RNA, either shRNA or siRNA, to reversibly silence specific genes.

For ectopic expression, several techniques can be used to introduce novel genetic sequences into the mouse genome, including homologous recombination into a specific locus in the mouse genome, or random integration of transgenes through injection into an embryo. These techniques have been used to express mutant forms of genes, over-express genes, mis-express genes in different cell types, express exogenous genes such as GFP or LacZ reporters, or express toxins to kill specific cell types [2]. Several methods have been used to control the specificity of the expression of the transgenes (Figure 1). The transgene can be generated downstream from a defined promoter, and thus the expression will be controlled by the specificity of the promoter. The transgene can be generated downstream from a strong promoter and a stop cassette that is flanked by lox-p sites (lox-stop-lox). In this case, the stop cassette will inhibit the expression of the gene, unless the cassette is excised by Cre recombinase, and thus the onset of the expression is controlled by the Cre recombinase, but expression is controlled by the upstream promoter once the lox-p sites have been removed. Often the lox-stop-lox transgene cassette is inserted in the ROSA locus by homologous recombination. The *ROSA* locus has been shown to ubiquitously express genes, and thus inserting a lox-stop-lox reporter cassette into this locus marks all cells downstream of the cell in which the cre-recombinase excision has occurred. Zambrowicz *et al*. showed that insertion of the *β*-*galactosidase* gene at the *ROSA* locus in mice induced a broad β-gal activity throughout the body [3].

Another common technique utilized is to generate the transgene downstream of the tetracycline response element (TRE) (Figure 1). The TRE element promotes the expression of genes when the reverse tetracycline transactivator (rtTA) and doxycycline are both present. Therefore, spatial control of gene expression can be achieved by the expression of rtTA in response to cell specific promoters, and temporal expression can be reversibly achieved by altering the levels of doxycycline in the diet. This method can also be used with a tetracycline transactivator (tTA) that induces expression from the TRE reporter when doxycycline is removed from the diet. Additionally different methods of viral infection, electroporation, liposomal transfer and other techniques have been utilized to deliver genetic material to specific cells in mice.

## Mouse models used to study the BBB

### Targeting endothelial cell function

#### Tight Junctions

**Figure 2:**
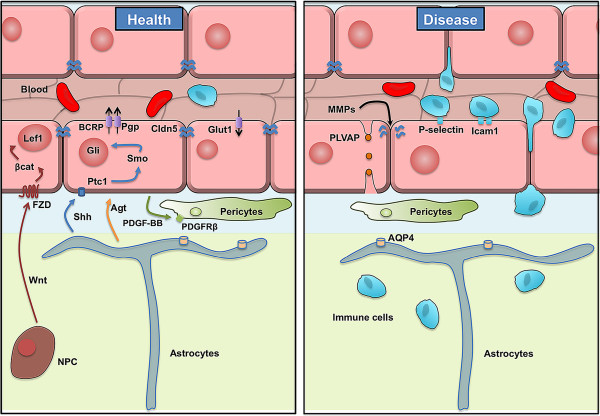
**Schematic representation of the BBB in health and diseases.** Schematic representation of the blood–brain barrier in health (left side) and during pathological breakdown during injury and disease (right side). CNS endothelial cells (pink) form BBB properties, and interact with cells in the blood (RBC-red, leukocyte-blue) and in the neural tissue (pericyte-green, astrocytes-taupe). Many of the BBB properties are altered during diseases such as stroke and MS.

CNS ECs differ from ECs in non-neural tissues in that they are held together by TJs which greatly restrict the paracellular movement of molecules and ions between the blood and brain. Most of the TJ proteins have been identified by work on epithelial cells, which has demonstrated that TJs are formed by a series of transmembrane proteins, including the claudins [4,5], occludin [6] and junctional adhesion molecules (JAMS) [7] which are linked to the cytoskeleton and adherens junctions by adaptor molecules including ZO-1, ZO-2, Cingulin and others. In particular, claudins are a family of >20 tetraspanin genes in mammals and expression of specific claudin family members in different cellular barriers is thought to be important for the specific paracellular physiology of the barrier [8]. Claudin 5 has been identified as a major constituent of the TJs of CNS ECs (Figure 2). Nitta and colleagues have generated *Cldn5* knockout mice [9]. These mice die at birth, and embryos have been shown to have a size selective leakiness of the BBB, with leaks to small molecules (up to 800 Da) but not large molecules (serum albumin, 68 kDa and microperoxidase, 1.9 kDa). The BBB TJs look ultrastructurally normal in the absence of claudin 5 suggesting that other TJ proteins are sufficient to form the structural junctions. In fact, claudin 3 and 12 have been identified as being expressed by CNS ECs [10,11]. The *Cldn5* knockout mouse strain is a complete knockout and thus this mouse model cannot be utilized to study the cell autonomous action of claudin 5 in CNS ECs.

Pfeiffer and colleagues have developed tools utilizing the tTA/TRE system to ectopically express claudin 1 in ECs [12]. This group used a double transgenic model in which the tTA was expressed from the Tie2 pan-endothelial promoter, and claudin 1 was expressed from the TRE promoter, therefore claudin 1 would be ectopically expressed in ECs if the mouse diet lacks doxycycline. They used this model to express claudin 1 in ECs during neuroinflammation in experimental autoimmune encephalomyelitis (EAE), a mouse model of multiple sclerosis (MS) [13]. During EAE there is a breakdown of the BBB which allows the entry of immune cells and molecules into the CNS which attack the CNS myelin causing damage to the CNS. This group showed that ectopic expression of claudin 1 seals the BBB during this disease and lessens the symptoms of EAE.

Occludin is a tetraspanin found at TJs in all epithelial cells, and has been identified as being expressed by CNS ECs [6,14]. Saitou and colleagues have generated *Ocln* knockout mice, which are viable but the males are infertile [15]. The TJs in the epithelial cells and CNS ECs appear ultrastructurally normal in the *Ocln* knockout mice, and the measurements of the electrical resistance of the intestinal epithelial cells is also unperturbed, suggesting that TJs form a functional barrier in the absence of occludin. Interestingly, the *Ocln* knockout mice have calcification of the brain suggesting that there could be specific defects in the regulation of the paracellular movement of calcium.

#### Transcytosis

Transcytosis is the process by which a vesicle is trafficked through the cell from one surface to the other and it can be accomplished through: a receptor-mediated mechanism by specific binding from a ligand to its receptor, by a non specific uptake called pinocytosis, or an adsorptive-mediated mechanism initiated by electrostatic forces between the negatively charged ECs membrane and positively charged proteins. CNS ECs undergo extremely low rates of transcytosis compared with ECs in non-neural tissues, which greatly limits the transcellular movements of hydrophilic molecules between the blood and the brain. An increase in the number of transcytotic vesicles in CNS ECs has been observed in several diseases in which there is breakdown of the BBB [16-18]. The vesicle-mediated transport is primarily mediated through caveolin based vesicles ([19] for review). Several groups have made *Cav1* knockout mice, including a caveolin-1 conditional lox-p flanked allele, however the complex phenotype in the mice throughout the vascular network makes it very difficult to study the role of caveolin-1 specifically at the BBB [20-25]. Plasmalemmal vesicle-associated protein-1 (PLVAP) is a transmembrane protein associated with the caveolae of fenestrated microvascular ECs [16]. In rodents, PLVAP expression is enriched in non-CNS ECs compared to CNS ECs [26]. Interestingly, during diseases like ischemia/stroke, acute ischemia, tumors or diabetic retinopathy, PLVAP1 is upregulated in CNS ECs (Figure 2) [18,27,28]. Mouse ES cell lines have been targeted for *PLVAP1*, for both knockout and conditional alleles, however mutant mice have not yet been described.

#### Efflux transport

**Figure 3:**
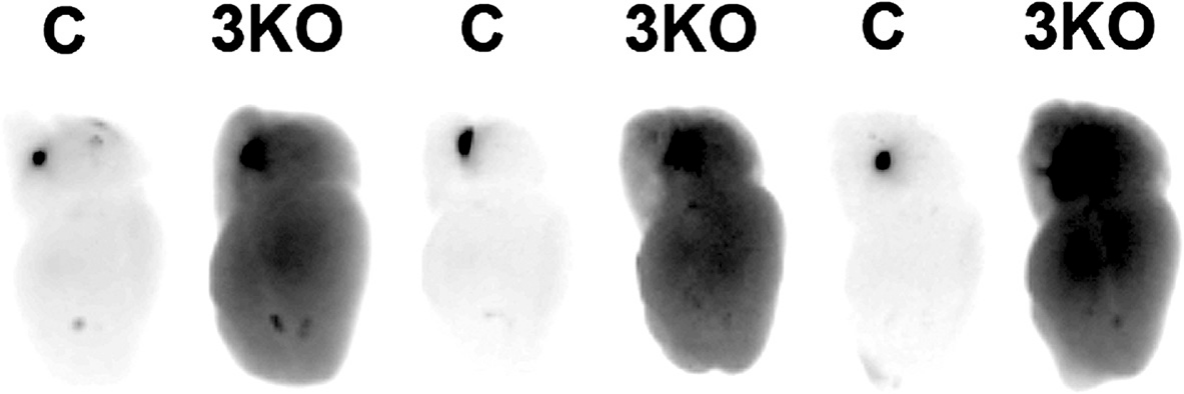
***Abcb1a*/*Abcb1b*/*Bcrp *triple knockout mice have a leaky BBB to Rhodamine123.** Adult wild type (C) or *Abcb1a*/*Abcb1b*/*Bcrp* triple knockout mice (3KO) were given an intravenous injection of Rhodamine123 (10 ng). After 1 hour the blood compartment was removed by transcardiac perfusion with PBS, the brains were removed, hemisected down the midline and imaged for Rhodamine123 fluorescence with a Fujifilm imager LAS 4000. Figure 3 represents an image of the hemisected brains with the cerebellum at the top of the image and frontal cortex towards the bottom of the image. More Rhodamine123 (dark color) was observed in *Abcb1a*/*Abcb1b*/*Bcrp* triple knockout mice compared with littermate controls. To demonstrate the consistency of the phenotype between different animals, 3 wild-type and 3 *Abcb1a*/*Abcb1b*/*Bcrp* triple knockout mice brains were utilized.

CNS ECs express efflux transporters to eliminate potential toxins from the CNS. These include members of the ATP-binding cassette (ABC) transporters, which utilize the hydrolysis of ATP to transport a wide variety of substrate molecules against their concentration gradient. In particular, CNS ECs express P-glycoprotein (Pgp/Mdr1/Abcb1) and the breast cancer resistance protein (Bcrp/Abcg2) (Figure 2), each of which has diverse but potentially overlapping substrate specificity [29-31]. The mouse genome contains two Pgp genes: *Abcb1a* and *Abcb1b*. Several mouse lines are available for studying *Abcb1a*, including targeted gene disruption (*Abcb1a*^tm1bor^), a Cre/lox regulated luciferase targeted into the *Abcb1a* locus (*Abcb1a*^tm1Kane^) and a spontaneous mutation (*Abcb1a*^mds^) that has a long terminal repeat of the ecotropic murine leukemia virus inserted into an intron [32-34]. Mice homozygous for *Abcb1a*^tm1bor^ allele have a BBB that is more permeable to specific molecules including different xenobiotics and drugs [32]. Because Pgp has 2 isoforms, Doran and colleagues generated a double knockout mouse of *Abcb1a* and *Abcb1b* to study drug delivery [35]. Finally, as Pgp and Bcrp can transport some of the same substrates, the *Abcb1a*/*Abcb1b*/*Bcrp* triple knockout mouse was genetically engineered [36] and found to have a leaky BBB for many lipophilic xenobiotics, including rhodamine123, compared to their wild type (WT) littermates (Figure 3). These mice are very useful to study brain neuroprotection and neurotoxicity [37]. However one has to keep in mind that in these widely-used knockout strains, the efflux transporters are deleted in every cell throughout the body, and not specifically in CNS ECs.

#### Influx transport

CNS ECs express a series of solute transporters that transport specific nutrients into the brain including: glucose (*GLUT1*/*Slc2a1*), lactate (*MCT1*/*Slc16a1*), amino acids (*Slc7a1*, *Slc7a5*) and others (for review see [38]). Mouse null knockout models have been generated for a number of different transporters, however these often have phenotypes throughout the organism, as diverse cell types often require transport of these nutrients. For instance, *Slc7a1* knockout mice die in the neonatal period with severe anemia [39], whereas *Slc7a5* knockout mice display embryonic lethality [40]. Therefore, for many of these solute carriers it may be critical to develop conditional alleles to specifically study their function at the BBB.

GLUT1 has been largely studied for its role in delivery of glucose to the CNS (Figure 2). Glucose is the primary source of energy for the brain, and human *GLUT1* deficiency results in an epileptic syndrome. A *Glut1* knockout allele has been generated with targeted disruption of the promoter and exon1 of the gene [41]. Mice homozygous for this allele die during embryogenesis with pleiotropic phenotypes, whereas mice heterozygous for this *Glut1* mutation display a 66% decrease of GLUT1 protein in the brain, and have similar symptoms as the *GLUT1* deficiency syndrome found in humans including epileptic events and impaired motor activity. A second group used a gene-trap method to disrupt the *Glut1* locus [42]. For this gene-targeting model, the trapping vector contains a splice acceptor site along with the neomycin coding sequence and a polyadenylation sequence, and thus when inserted into the *Glut1* locus, the upstream *Glut1* sequence is spliced to the trapped sequence forming a truncated mRNA. Following the polyadenylation sequence, the vector also contains a Bruton’s tyrosine kinase (*btk*) gene with a splice donor site, and thus a fusion mRNA is expressed with the Btk mRNA fused to the downstream *Glut1* sequence. Mice homozygous for this gene trap demonstrated embryonic lethality, whereas heterozygous mice displayed no phenotype. The difference in phenotype observed between the *Glut1* heterozygotes generated from these two studies may be due to the differing affects of the targeting on gene expression, or compensation from other transporters such as the monocarboxylic acid transporters MCT1 and MCT2. A lox-p flanked conditional *Glut1* allele has now been generated that can be utilized to study GLUT1 in specific cell types, such as CNS endothelial cells [43]. In addition, Heilig and colleagues developed a transgenic mouse line in which the expression of antisense-GLUT1 sequence was driven from the *b*-*actin* promoter in order to knockdown the glucose transporter during development [44]. Hemizygous or homozygous embryos for this transgene display reduced glucose uptake.

#### Leukocyte adhesion

The healthy CNS has an extremely low level of immune surveillance with the almost complete absence of subsets of leukocytes including neutrophils, T-cells and B-cells, however changes to the BBB during diseases including multiple sclerosis (MS), stroke, and neurodegenerative disorders can allow entry of immune cells into the CNS which is an important component of the pathogenesis of these diseases. Entry of immune cells into a tissue is a multi-step process that involves the binding of a series of adhesion molecules expressed on the immune cells to adhesion molecules on the post-capillary venule ECs [45]. This process involves tethering of the leukocyte to the endothelium, rolling along the endothelium, activation of the leukocyte, firm adhesion to the endothelium, and transmigration between or through the ECs. Several adhesion molecules on the endothelium have been identified, including P-Selectin and E-Selectin for rolling adhesion, and Icam1 and Vcam1 for firm adhesion (Figure 2). The expression of these adhesion molecules is low in healthy CNS ECs, but can be dramatically upregulated during injury and disease.

Several genetic mouse models have been developed to study leukocyte adhesion and transmigration in different models for CNS diseases. This review focuses on the different genetic manipulations of the BBB, however here we briefly introduce a few of the disease models in which BBB dysfunction is commonly analyzed. MS is an inflammatory demyelinating disease of the CNS with numerous neurological symptoms that can lead to physical and/or cognitive disability. The most common animal model of MS used to study its immune and inflammatory components is called experimental autoimmune encephalomyelitis (EAE). The disease is induced by immunization of a myelin peptide (such as MOG) emulsified in an adjuvant that will induce the inflammatory response, with pertussis toxin often used to facilitate the induction of EAE. A stroke is a loss of function of brain cells caused by an alteration of blood flow, most of the time in a cerebral artery, that limits the supply of oxygen and glucose to specific regions of the CNS (ischemia). Many studies focussing on ischemic stroke use the middle cerebral artery occlusion (MCAO) model. In this model the middle cerebral artery is ligated for minutes or hours which then may or may not be followed by a release of the ligation leading to reperfusion of the injured tissue. In addition, mouse models for different neurodegenerative diseases with BBB dysfunction are often utilized including amyotrophic lateral sclerosis, Alzheimer’s disease and Parkinson’s disease.

Knockout mice for *P*-*selectin* or its ligand *PSGL*-*1* have been used to study leukocyte migration in neurological disease. In a stroke model, *P*-*selectin* knockout mice demonstrate decreased BBB breakdown to gadolinium and IgG as well as less infiltrating polymorphonuclear leukocytes [46]. In an epilepsy model, *PSGL*-*1* knockout mice displayed attenuated seizures suggesting that leukocyte CNS infiltration was an important component of the disease [47]. On the other hand *P*-*selectin* knockout or *PSGL*-*1* knockout did not affect the disease severity in several different EAE models [48-50]. *E*-*selectin* knockout mice have also been generated and have defects in neutrophil infiltration in different tissues that are exacerbated when combined with *P*-*selectin* knockout mice [51], but in an EAE model deficiency in *E*-*selectin* does not affect the progression of the disease [50]. However *E*-*selectin* knockout mice have not been extensively utilized to study neurological disease.

Firm adhesion is mediated through binding of CD11/CD18 and αVβ1 integrins on leukocytes to EC Icam1 and Vcam1, respectively. Icam1 is an adhesion molecule composed of repeating immunoglobulin-like domains, and several different gene disruption strategies have been engineered to develop *Icam1* knockout mice including the *Icam1*^tm1Jcgr^ allele which disrupts exon 4 (Ig domain 3) [52], the *Icam1*^tm1Bay^ allele which disrupts exon 5 (Ig domain 4) [53], or *Icam1*^tm1Alb^ allele which deletes the entire coding sequence [54]. Whereas the *Icam1*^tm1Alb^ allele lacks all Icam1 isoforms, the *Icam1*^tm1Jcgr^ and *Icam1*^tm1Bay^ alleles each produce specific alternatively spliced Icam1 isoforms. Therefore comparing the phenotype of each knockout mouse can identify roles for different Icam1 isoforms. Interestingly, during EAE, *Icam1* null mice (harboring *Icam1*^tm1Alb^ alleles) have attenuated disease symptoms [55], whereas mice with *Icam1*^tm1Bay^ alleles had worse EAE symptoms [55,56]. These data suggest that specific isoforms of Icam1 may have differing functions in regulating neuroinflammation. Furthermore, Hu *et al*. used adoptive transfer of encephalitogenic T-cells from wild type to mutant strain or vice versa to determine the cell autonomous function of these isoforms [55]. *Vcam1* knockout mice have also been generated and die during embryogenesis [57], however several lox-p flanked alleles have been generated in order to study the postnatal and cell specific function of Vcam1 [58-61].

Several adhesion molecules have been identified that regulate the adhesion of specific subsets of leukocytes to the endothelium. For instance, Th17 cells express MCAM which binds to laminin 411 on the EC basement membrane [62], CD4^+^ lymphocytes express CD6 which binds to EC ALCAM [63], and ninjurin-1 on myeloid cells homotypically interacts with ninjurin-1 on inflamed ECs [64]. Mouse knockouts for *Alcam*[65] and *Mcam* (Mcam^tm1Lex^) have been generated, however these knockout models have not been extensively utilized to study neurological disease.

#### Matrix metalloproteinases

Matrix metalloproteinases (MMPs) are secreted zinc-dependent endopeptidases that can degrade components of the extracellular matrix. Twenty-eight MMP family members have been reported so far, but in particular MMP2, MMP9 and MMP12 have been suggested to play a key role during CNS disease by disrupting the BBB. By deleting *Mmp2* or *Mmp9*, researchers have found that the mice were protected after ischemia/reperfusion with attenuated inflammation of the brain [66-68]. Recently *Mmp12* deficient mice have been studied during Theiler’s murine encephalomyelitis (TME), a virus-induced model of MS [69], and lack of MMP12 produced a reduction in macrophage infiltration and demyelination with an intact BBB (Figure 2). It will be interesting to define the role played by each MMP during different neurological disorders.

#### CNS angiogenesis and BBB development

BBB development involves the complex interaction of CNS cells with different neural and immune cells. The process of BBB regulation begins with induction signals as ECs invade the CNS during development and continues with maintenance signals throughout life and ageing. Here we discuss selected genetic models that have been used to dissect this process including manipulating pathways that affect angiogenesis (VEGF, Notch), CNS-specific angiogenesis (Wnt/β-catenin, Gpr124), BBB maintenance (Shh, Agt) and BBB ageing (ApoE) (Figure 2).

##### *VEGF*:

Vascular endothelial cell growth factor (VEGF) was first shown in 1989 to specifically activate EC proliferation [70]. In mammals, 5 different VEGF molecules have been discovered (VEGF-A, Placental Growth Factor (PIGF), VEGF-B, VEGF-C, VEGF-D), as well as three distinct receptors (VEGF-R1/Flt-1, VEGF-2/Flk-1/Kdr and VEGFR-3/Flt-4) [71]. Mice knockout models have been developed for each ligand and receptor, and conditional alleles have been generated for VEGF-A, Flt-1 and Kdr. VEGF-A, a paracrine factor, and its endothelial receptors Flt-1 and Flk-1 are the most extensively characterized members, and mice homozygous for null alleles of each gene are embryonic lethal [72-75]. By studying mice with a lox-p flanked conditional mutant allele for *VegfA* in conjunction with a Nestin-Cre allele to delete *VegfA* in the neural precursors, it was demonstrated that the level of vascularity in the developing brain is dependent on levels of VEGF-A [76,77]. PGF has been less studied than VEGF-A but it has been shown to play a critical role in vessel stabilization under pathological events [78], and recently Freitas-Andrade *et al*. developed a mouse deficient for *Pigf*[79]. This group showed that after a hypoxic event the *Pigf* knockout mouse displayed a delayed angiogenic response and an increased BBB permeability to endogenous fibrinogen. All the studies using mouse knockout models for VEGF family members point out its critical role in angiogenesis throughout the body including the CNS.

##### *Notch*:

Notch signaling is an evolutionarily conserved mechanism that is best known for its function in cell-fate decision in various tissues [80]. In mammals four Notch receptors and five ligands have been identified with diverse expression patterns [81]. In mouse embryos Notch1 and Notch4 are predominantly expressed on the arterial endothelium. When the *Notch1* gene is inactivated specifically in the endothelium, mutant embryos die at embryonic day E10.5 with normal vasculogenesis but important defects of angiogenesis [82]. *Notch4*-deficient mice exhibit normal development without any vascular abnormality [83]. However, the double *Notch1*/*Notch4* mutant mice have vascular defects more severe than the single *Notch1* mutant suggesting overlapping functions of both receptors during development [83-85]. By using the Tie2-tTa system coupled with the TRE-caNotch4, a constitutively active Notch4 mutant was specifically expressed in the endothelium of postnatal mice [27]. These mice show abnormal connections between arteries and veins associated with ectopic expression of the arterial marker ephrin B2 in veins. Activation of constitutively active Notch4 in the blood vessels of the developing mouse brain induces vessel enlargement followed by hemorrhages in the cerebellum and the neocortex, neurological damage and death [86].

Four of the 5 known Notch ligands (Delta-like 4 (Dll4), Dll1, Jagged1 and Jagged2) are specifically localized in arterial but not in the venous endothelium [81,83,87]. During early vascular development, Dll4 share the same expression pattern as Notch1 and Notch4 [83]. Homozygous and heterozygous gene inactivation of *Dll4* leads to embryonic lethality in several mouse strains between embryonic days E9.5 and E10.5 due to severe vascular deffects [88,89]. However, in the outbred ICR strain, the heterozygous mutation leads to limited embryonic lethality [88,89]. Therefore, by using *Dll4*^+/−^ mice retinas in the ICR strain, it was shown that absence of one *Dll4* allele leads to an increase of endothelial tip cells that sense and respond to guidance cues during angiogenesis [90-92]. Moreover, Hellstrom *et al*. demonstrated a similar phenotype when *Notch1* gene was inactivated specifically in ECs, suggesting that during angiogenesis, signaling through Dll4/Notch1 is responsible for the regulation of endothelial tip cell formation [90] in response to VEGF [90-92]. Although Dll1 is not involved in arterial cell fate, it has recently been shown to be required for maintaining arterial identity by using a transgenic mouse line that inducibly deletes Dll1 in endothelial cells [93].

##### *Wnt/ β-catenin*:

Several groups have demonstrated that Wnt/β-catenin signaling is specifically activated in CNS ECs during development and is required for angiogenesis into the CNS as well as development of the BBB [94-96]. Wnts are secreted ligands that bind to Frizzled receptors at the cell surface, which leads to inactivation of a protein complex that degrades β-catenin. Stabilized β-catenin is then able to translocate to the nucleus and activate transcription along with Lef1/Tcf complexes [97]. A number of different genetic mouse models have been used to analyze different aspects of Wnt/beta-catenin signaling at the BBB [94,96]. Several transgenic Wnt reporter mouse lines have been generated that have cDNA encoding a reporter protein (LacZ, GFP) downstream of Wnt responsive DNA elements such as TCF binding sites (for review see [98]). These Wnt reporter mice, including TOP-Gal, BAT-Gal and TOP-Flash, have been used to identify Wnt activity in CNS ECs (for review see [98]).

A number of mouse models have been developed to target β-catenin activity. Several groups have developed endothelial-specific *β*-*catenin* knockout mouse lines using Tie2-Cre and *β*-*catenin* lox-p flanked alleles [94,96,99]. This model has demonstrated that endothelial β-catenin is required for angiogenesis into the CNS [94,96,99], and for the expression of BBB-specific transporters such as GLUT1 [94-96]. There are several caveats to this approach of inhibiting Wnt signaling. First, Tie2-Cre/β-catenin mutants die during early embryogenesis, and so while they have been effective for studying early angiogenic events, they have been less successful for studying BBB maintenance. To address this concern, Liebner and colleagues utilized a *Pdgfb-CreERT2* allele to delete lox-p flanked *β*-*catenin* alleles in endothelial cells at postnatal ages, to demonstrate that β-catenin was required for sealing off the BBB [95]. Second, Tie2-Cre is also active in hematopoietic lineage cells [100], so each time this line is used one has to be sure that phenotypes are not due to changes in blood cells. Third, β-catenin is not only required for transduction of canonical Wnt signaling, but is also a component of the adherens junctions, and thus it is difficult to gene-rate conclusions specifically about Wnt signaling from β-catenin mutants. In addition to conditional knockout strategies, transgenics have been used to generate gain of function β-catenin mouse alleles by generating a transgenic *β*-*catenin* with exon3 flanked by lox-p sites, and thus when exon3 is removed the mutant *β*-*catenin* is constitutively active. Using *Pdgfb-CreERT2*/*β*-*catenin* loxp-exon3-loxp mice, Liebner and colleagues were able to activate β-catenin in the embryo and observe precocious BBB maturation [95].

Several genetic models have been utilized to inhibit other aspects of Wnt signaling, including analysis of *Wnt7a*/*Wnt7b* double knockout mice as these are the Wnts with the broadest expression pattern in the developing CNS [94,96]. These mice die at embryonic day 12.5 and have angiogenesis deficits in the CNS, vascular malformations and hemorrhage. Due to the early embryonic lethality of *Wnt7b* mutants, Stenman and colleagues used a conditional approach by generating a mouse line with null alleles of *Wnt7a* and loxp flanked *Wnt7b* alleles in conjunction with a Nestin-Cre to delete *Wnt7b* in the developing neuroepithelium [96]. These mice lived longer than the complete double knockout of *Wnt7a*/*Wnt7b* mice, and thus gave vital information about the role of Wnts in regulating CNS vessel development. In addition, there are many different positive (Wnt, β-catenin, Tcf) and negative (Axin2, Apcdd1, APC, Dkk, sFRP) regulators of Wnt signaling, and mouse knockout and over-expression alleles have been generated for many of these (reviewed [97]). Recently, Tam *et al*. showed the critical role of both TROY and DR6 for CNS angiogenesis as downstream target genes of the Wnt/β-catenin signaling [101]. They showed that *DR6* mutant mice display a lower density of brain vasculature and a leaky BBB for Evan’s blue dye, with a lower amount of ZO1 protein in adult mice. In mouse embryos they observed hemorrhages in the forebrain with a leaky BBB for sulfo-NHS-biotin, coupled with a lower vascular density in the hindbrain. To determine the endothelial specificity of these phenotypes, Tam *et al*. generated a mouse with Tie2-Cre and exon2 *DR6* lox-p flanked alleles, and described similar phenotypes to full knockout mice suggesting that DR6 expression is required specifically in endothelial cells. *Troy* knockout mice display a mild leakage of the BBB for Evan’s blue.

##### *Gpr124*:

Recently, several groups have generated mouse knockouts for *Gpr124*, which displayed a disruption of angiogenesis in the forebrain and ventral spinal cord with localized malformations and hemorrhages, demonstrating that this G-protein coupled receptor was required for CNS-specific angiogenesis [102-104]. Using mice with lox-p flanked conditional alleles and Tie2-Cre transgenes, it was demonstrated that Gpr124 function is specifically required in the ECs [102,104]. Interestingly, the phenotype looks similar to that observed in the *Wnt7a*/*Wnt7b* double knockout mice, however it remains unclear whether Gpr124 and Wnt signaling are connected.

##### *Hedgehog*:

The Hedgehog (Hh) family, first characterized in Drosophila [105], are secreted morphogens [106] that play a major role in development including neuronal guidance and angiogenesis [107,108]. Three members of the Hh family have been identified in mice: Sonic Hedgehog (Shh), Desert hedgehog (Dhh) and Indian hedgehog (Ihh). Shh acts by binding to Patched, which leads to de-repression of Smoothened (Smo) which activates genes through the transcription factor Gli [106]. Chiang and colleagues have generated a knockout mouse model for *Shh*[109]. *Shh* mutant mice display embryonic lethality with embryos having abnormal anatomy in several parts of the body including the brain and spinal cords. In the CNS, when Shh is overexpressed in the dorsal neural tube of embryos, *Shh* transgenic mice display a hypervascularization [110]. Alvarez and colleagues described how the Hh pathway contributes to the maintenance of BBB functions [111]. They showed that E13.5 embryos of the *Shh* knockout mice display a lower amount of junctional proteins in the brain capillaries than their WT littermates. To study the role of the Shh pathway specifically in ECs, they generated endothelial-specific *Smo* knockout mice by using a *Tie2-Cre* allele and a *Smo* lox-p allele. The BBB of the mutant mice is permeable to serum proteins, like fibrinogen, apolipoprotein B and immunoglubulins in E14 embryos and P19 mice and the BBB of adult mice is permeable to exogenous compounds. The BBB leakiness was explained by a significant decrease of several TJ proteins including claudin 3, claudin 5, occludin and ZO1 and a fragmented basement membrane. Moreover, Alvarez *et al*. demonstrated that Shh plays a key role in the regulation of the pro-inflammatory response during EAE. Altogether, these data suggest two major roles of the Hh pathway by regulating BBB function and protecting the brain from inflammation.

##### *Renin-angiotensin*:

In the brain, the renin-angiotensin system controls cerebral blood flow, memory and BBB function (for review see [112]). Astrocytes express angiotensinogen (Agt), a precursor of angiotensins I-IV (Ang). In a mouse model deficient for *Agt*, Kaninuma and colleagues demonstrated that two weeks after a brain cold injury, the knockout mice still display a leaky BBB compared to their WT littermates whose BBB was repaired [113]. This phenotype was less critical when AngII or AngIV was given to the *Agt* deficient mice suggesting their critical involvement in vascular repair after an injury. Moreover, the *Agt* mutant mice have a leaky BBB for endogenous serum plasminogen and albumin and express less occludin at the EC TJs [114].

##### *ApoE*:

Several groups have identified apolipoprotein E (*apoE*) as a key regulator of BBB leakiness [115,116]. ApoE is mainly expressed by glial cells in the CNS where they mediate transport uptake of lipoproteins [117]. The knockout mouse of *ApoE* has a leaky BBB in 2-week old and adult mice [115,116] that increases during ageing [118]. The pericytes of the *ApoE* knockout mice have a higher amount of cyclopilinA (CypA), a proinflammatory cytokine, and nuclear translocation of the NF-κB factor that transcriptionally activates MMP9, which correlates with a decrease of EC TJ proteins such as ZO1, occludin and claudin 5 at the BBB [115]. Moreover pericyte coverage of ECs is decreased as well as the length of the capillaries. By generating a double knockout mutant that targets *ApoE* and *CypA* genes, Bell *et al*. demonstrated a rescue of the aforementioned phenotypes suggesting that the over-expression of cypA in the *ApoE* knockout mice was important for the BBB dysfunction [115].

### Targeting pericyte function

Pericytes are mural cells that incompletely surround the abluminal surface of the capillary endothelium (Figure 2). These cells are derived from the neural crest and regulate angiogenesis, vascular remodeling, leukocyte trafficking and the formation and function of the BBB [119-121]. The binding of the ligand platelet-derived growth factor-BB (PDGF-BB) to the platelet-derived growth factor receptor β (PDGFRβ) is required for the generation and recruitment of pericytes to CNS vessels as *Pdgfb* knockout mice and *Pdgfrb* knockout mice completely lack CNS pericytes [122,123]. These mice have altered vascular patterning, dilations in the microvasculature, and form micro-aneurysms that occasionally hemorrhage. The ability to study the role of pericytes in BBB function is limited in both *Pdgfb* knockout and *Pdgfrb* knockout mice as they die shortly after birth, however the *Pdgfrb* knockout mice have been utilized to demonstrate that pericytes are required for BBB formation during embryogenesis and that they regulate the BBB by inhibiting the expression of EC genes that would make the vessels leaky [119,120].

Several groups have developed genetic models which decrease PDGFBB signaling through PDGFRβ without completely abolishing it. Tallquist and colleagues have generated a series of hypomorphic alleles of *Pdgfrb* in which different numbers of tyrosine residues, which are normally auto-phosphorylated upon ligand binding, are mutated to phenylalanine residues [124]. Using different combinations of these hypomorphic alleles, they were able to generate mice with different numbers of pericytes. These mice have been used to demonstrate that the relative number of pericytes is important for the permeability of the BBB during development [120]. Furthermore, Bell and colleagues used this model to demonstrate that during ageing there was reduction in capillary perfusion and BBB breakdown that led to neural degeneration [125]. Interestingly, whereas there are BBB defects in this model during development and ageing, the BBB appears somewhat normal during adulthood. Several genetic models have also targeted the ligand to attenuate PDGFB signaling. Lindblom and colleagues developed mice in which the retention motif of PDGFB was deleted, such that PDGFB binding to extracellular matrix heparan sulphate proteoglycans was disrupted, and mice homozygous for this allele had 26% of the pericyte coverage of WT mice [126]. In addition, Armulik and colleagues generated mice that had lox-stop-lox human PDGFB transgene at the *ROSA* locus, and thus could ectopically express human PDGFB in ECs by using a Tie2-Cre mouse line [119]. Using a *Pdgfb* null knockout mouse as a background, they could express one or two alleles of the human *PDGFB* and thus generate mice with attenuated signaling that had 40% and 72% the number of pericytes of WT mice. Using these lines, Armulik *et al*. demonstrated that pericytes were required for BBB function in adults, and did so by inhibiting the rates of transcytosis [119]. One interesting point is that there is a slight difference in the phenotype of the mice when signaling is attenuated by targeting *Pdgfb* or *Pdgfrb*. The *Pdgfrb* hypomorphic mice have a leaky BBB during development and ageing but relatively normal BBB as adults, whereas the models attenuating *Pdgfb* have a leaky BBB as adults. Several reasons could lead to these differences including: strain of mice, environment of mice, total numbers of pericytes, signaling of PDGFB through multiple receptors, or localization of signals.

Goritz and colleagues utilized a specific GLAST-CreER/RosaYFP line to fluorescently label a specific subtype of pericytes, which they termed type A pericytes [127]. To accomplish this, they utilized a mouse in which a lox-stop-lox YFP cassette was introduced into the *ROSA* locus by homologous recombination, and thus the YFP reporter would be expressed in cells following the Cre-recombinase mediated excision of the stop cassette. Using the GLAST-CreER line, they demonstrated that upon injection of tamoxifen in adults, the YFP reporter was expressed in the spinal cord in a subset of pericytes. They then demonstrated that following a spinal cord injury these type A pericytes migrated to the site of injury and formed the scar tissue. This group also used a Glast-CreER/RASless mouse line to inhibit cell division of the type A pericytes in the spinal cord injury model [127]. Rasless mice have null alleles for *H*-*Ras* and *N*-*Ras* and have *K*-*Ras* alleles flanked by lox-p sites. The mice are generally normal, except cells lack the ability to divide if Cre-recombinase mediated mutation of *K*-*Ras* occurs. Using the Glast-CreER/RASless mouse line coupled with tamoxifen injections in the adult, they were able to generate mice in which type A pericytes developed normally (as the CreER only excises the conditional allele upon tamoxifen injection in the adult), but failed to divide in the adult following a spinal cord injury model. This group showed that division of type A pericytes is required for scar formation following spinal cord injury.

In addition Li and colleagues manipulated TGF-β signaling in ECs to generate a mouse model that had deficits in endothelial-pericyte interactions [128]. This group generated a CNS endothelial conditional mutant of *Smad4*, a downstream mediator of TGF-β signaling, by utilizing lox-p flanked *Smad4* alleles and a SP-A-Cre mouse line. They demonstrated that disruption of *Smad4* in CNS ECs led to a mouse with defective pericyte coverage, intracranial hemorrhage and BBB breakdown.

### Targeting astrocyte function

Astrocytes are a major glial cell type in the CNS that send out highly ramified processes that ensheath both synapses and blood vessels (Figure 2). It is thought that in the rodent brain a single mature astrocyte can cover a space between 20,000 and 80,000 μm^3^ and contacts approximately 100,000 synapses and ensheaths one or two capillaries [129,130]. Astrocytes play an important role in regulating neuronal metabolic homeostasis, synapse formation, neurotransmitter processing, as well as coupling neuronal function with cerebral blood flow (for review see [131]). Transplantation studies and *in vitro* studies have suggested that astrocytes are important regulators of BBB function. When isolated from the brain, ECs lose their BBB properties as shown by a decrease in their trans-endothelial electrical resistance (TEER) [132]. When co-cultured with astrocytes or astrocyte-conditioned media their TEER increases significantly, suggesting that astrocyte-secreted factors are involved in activating the barrier properties of the BBB [132,133].

Several genetic models have been developed that manipulate astrocyte function (reviewed by Pfrieger and Slezak 2012 [134]). To selectively ablate astrocytes, several groups have induced ectopic expression of the herpes simplex virus thymidine kinase (HSV-TK) in astrocytes under control of either the human *GFAP* promoter or the murine *Gfap* promoter [135-137]. On its own HSV-TK does not affect cell viability. However, the enzyme converts ganciclovir into ganciclovir monophosphate, a nucleotide analogue which disrupts DNA replication. Therefore, cell division can be inhibited by addition of ganciclovir to HSV-TK expressing cells [138]. Delaney and colleagues used ganciclovir to inhibit cell division of GFAP-positive cells in neonatal GFAP-HSV-TK mice, and demonstrated that astrocyte reduction in newborn pups results in ataxia, neuronal excitotoxicity and a disorganization of Purkinje cells and radial glia [135].

Due to the wide scale effects of disrupting cell division in all astrocytes, Tsai and colleagues recently developed methods to deplete specific domains of astrocytes [139]. To accomplish this they generated a transgenic mouse line such that a lox-eGFP-stop-lox-Diptheria toxin-A (DTA) was expressed under the control of an astrocyte-specific *Aldh1L1*-promoter. In this mouse, eGFP is expressed in astrocytes, however following Cre mediated recombination of the lox-p sites, eGFP is no longer expressed, instead DTA is expressed which kills the cells. By mating this line with transgenic mouse lines in which Cre recombinase expression is driven from promoters that mark regionally-specific subsets of neural progenitors (Pax3-Cre, olig2-Cre). The authors were able to kill astrocytes in specific domains of the spinal cord by mating this line with transgenic mouse lines. For example, by mating the lox-eGFP-lox-DTA mice with the Pax3-Cre mice, the mutant line displayed variable perinatal lethality rates with a lower number of astrocytes in the dorsal spinal cord, but without an increase in their BBB permeability.

To study the role of reactive astrocytes during CNS pathology, Sofroniews’ group utilized the GFAP-HSV-TK mice combined with ganciclovir treatment to ablate dividing reactive astrocytes during disease models (for review see [138]). This group showed that reactive astrocytes were required for inhibiting neurite outgrowth, regulating neuronal survival and repairing the BBB following spinal cord injury [140]. During EAE, astrocytes form a scar that surrounds the blood vessels and mice with targeted ablation of proliferative astrocytes exhibit a much higher number of leukocyte infiltrations in the CNS parenchyma [141].

Aquaporin 4 (Aqp4) is a water channel protein mainly expressed in astrocyte endfeet that ensheath CNS blood vessels [142]. One function of Aqp4 is to facilitate water movement into and out of the brain. During a middle cerebral artery occlusion (MCAO), a mouse model of stroke, *Aqp4* deficient mice have a decreased cytotoxic cerebral edema and therefore an improved neurological outcome [143]. Saadoun *et al*. showed that *Aqp4* deficient mice have a morphologically and functionally normal BBB [144]. Therefore it appears that Aqp4 plays a key role in brain swelling during pathology, but not in normal BBB architecture.

### Imaging BBB function

Being able to visualize the movement of different cell populations *in vivo* in live mice is an important step in understanding how cells interact in physiological settings. This was made possible by the use of two-photon microscopy that allows brain imaging in living animals at a depth up to 1 mm. Several groups have utilized different genetic methods to label cells and proteins for imaging of CNS ECs and their interaction with the brain and immune cells. Transgenic mice with GFP expressed by the *Tie2* promoter have been utilized to label ECs *in vivo*. This technique has been used for microscopy, and we also have been able to purify brain ECs from these mice using fluorescence-activated cell sorting (FACS) and performed microarray analysis of their gene expression [26]. In addition, different subpopulations of ECs can be labeled for *in vivo* time-lapse imaging. Murphy and colleagues utilized *Ephrin*-*B2*-H2BGFP mice to visualize the nuclei of arterial ECs to examine the dynamics of cells during formation and regression of arterial venous malformations [145]. This mouse has a transgene of *histone*-*2B* fused to GFP that was inserted by homologous recombination into the first exon of the *ephrin*-*b2* gene, and thus a nuclear GFP was expressed from the *ephrin*-*b2* promoter [146].

Several different genetic techniques have been utilized to label different cell populations and analyze how they interact with the BBB. Davolos and colleagues developed methods to image the interactions of neurons and microglia with blood vessels in the spinal cord [147]. They performed intravenous (IV) injection of a fluorescent dye (rhodamine-dextran) into transgenic mice either with GFP inserted into the *Cx3cr1* locus to label microglia [148] or transgenic YFP-H line in which a *YFP* transgene is driven by the *thy1* promoter and thus expresses YFP in a subset of neurons [149]. The IV tracer labeled the blood inside the vessels and thus enables visualization of the interaction of microglia with the vessels over time. In the brain, Rangroo Thrane *et al*. used this technique to visualize the movement of eGFP-microglia during hepatic encephalopathy, a neuroinflammatory disease characterized by liver failure followed by an opening of the BBB [150]. Several groups have now utilized the microglia/macrophage reporter mice in which they express GFP from the *Cx3cr1* locus and RFP from the *Ccr2* locus, and thus have microglia labeled in green and macrophages labeled in red [151-153]. In addition different methods have been utilized to label astrocytes (see [134] for review) and pericytes *in vivo*[127].

To understand the interaction of auto-reactive T-cells with the BBB, an adoptive transfer model of EAE has been utilized with the injection of GFP-expressing MBP-reactive T-cells into mice [154,155]. This technique was utilized to examine the interaction of the T-cells with the vessels including arrest on the surface of the vessels, crawling against the blood flow, diapedesis and scanning of the abluminal surface for phagocytes [154]. Furthermore, this technique demonstrated that prior to entry into the CNS, the T-cells go into lung lymphoid tissues and lymph nodes to be activated. After their activation, the T-cells go back to the blood stream and migrate to the CNS parenchyma to induce clinical symptoms [155].

Other imaging methods, such as magnetic resonance imaging (MRI), positron emission tomography (PET) or X-ray microtomography, can be used to image blood vessels and BBB function in wild type mice as well as transgenic animals.

## Valuable tools available to study the BBB

A number of different tools have been developed in order to regulate gene expression in CNS ECs. To knockdown gene expression in ECs, several different models of the Cre/Lox system are available. One mouse line that has been generated is a Tie1-Cre [156]. Tie1 is a member of the Tie receptor family, and is essential for angiogenesis during embryogenesis. The *Tie1* promoter drives gene expression in ECs from embryonic day E10 until birth but also in a small part of hematopoietic cells and within some neuronal populations in the cortex and hippocampus [156]. In parallel, Tie2-Cre mouse lines were generated which is to date the most commonly utilized line for gene excision in ECs [100]. The *Tie2* promoter drives a similar expression pattern than *Tie1*, in all ECs with some hematopoietic cells, but it seems that it can start as early as embryonic day E8.5 [157]. When comparing both systems, some phenotypic differences can be seen during embryogenesis and were explained by the expression delay of Tie1 compared with Tie2 [158]. Although extensively used to delete lox-p alleles in ECs, several caveats arise from analysis of Tie2-Cre mice. First, Tie2 is turned on in hematopoietic precursors, and thus although Tie2 is no longer expressed in many blood cells, the Cre irreversibly deletes the lox-p flanked alleles in the precursors. Therefore, when analyzing phenotypes using conditional alleles in conjunction with Tie2-Cre, one must consider that the phenotype may arise from the function of the allele in ECs or hematopoietic lineage cells. Second, the Tie2-Cre can also excise lox-p flanked alleles in the female germline, and thus mating strategies must be used in which the Tie2-Cre with the lox-p flanked alleles are passed through male parents to ensure that a complete knockout is not generated.

Two different Flk-1-Cre lines are available, one that shows Cre expression in both vasculature and muscle lineages [159] whereas the second one does not have the muscle expression but seems to have a weak expression in quiescent endothelium [160]. A PECAM (CD31)-Cre has been generated to drive expression in endothelium, but is not extensively characterized [61]. A VE-cadherin Cre has also been generated [161]. The major interest of this Cre is that the promoter drives expression during embryogenesis as well as adulthood. Nevertheless, a strong VE-cadherin-Cre-driven expression starts later during embryogenesis than the Tie2-Cre system, around embryonic day E14.5 [161]. Recently, VWF-Cre and SP-A-Cre lines have been developed suggesting specific expression of Cre in CNS ECs, however these newly-generated lines have not been exhaustively studied [128,162].

Several attempts have been made in generating tamoxifen inducible Cre lines targeted specifically to ECs. A Tie2-CreERT2 transgenic mouse was genetically engineered [163] and shows a highly specific expression of lox-p flanked reporter transgenes in endothelial cells only when mice were treated with tamoxifen. In addition, two VE-cadherin-CreERT2 and a PDGFB-CreERT transgenic mouse lines were generated to express the tamoxifen-inducible CreERT(2) from EC promoters [164-167]. Several caveats have arisen with these systems. Firstly, whereas these transgenic alleles seem to efficiently excise lox-p flanked alleles if tamoxifen is given to embryonic or neonatal mice, the efficiency of recombination is often reduced during adulthood [166]. Furthermore, the timing of tamoxifen injections and the age of analysis has to be carefully controlled to determine if blood cells are also targeted.

In addition groups have generated Tie2-Tta or VE-Cadherin-tTA transgenic mouse lines in order to express TRE driven transgenes specifically in ECs [86,168]. One major advantage of the tTA/TRE system is that it is reversible, and thus by controlling the timing of doxycycline being fed to the transgenic mice it is possible to turn on and turn off the TRE driven transgenes. Additionally, Tie2-GFP mice have been utilized to visualize as well as purify ECs from the CNS [26,169].

To reduce the amount of pericyte coverage on the blood vessels, several groups have generated *Pdgfb* and *Pdgfrβ* deficient mice, as well as hypomorphic alleles of the ligand and receptor [119,126]. However, to date there are only a few mouse models to delete gene expression in pericytes. The most common line is the Pdgfrb-Cre [170] but the receptor is expressed by several mesenchymal cell types. Recently, Feng *et al*. developed a Ng2-CreERT to inducibly knockdown gene expression in pericytes, but in the CNS Ng2 is also expressed in oligodendrocyte precursor cells [171].

To deplete the brain of astrocytes, GFAP-HSV-TK and diptheria toxin systems have been generated [138,139]. To delete genes in astrocytes, there are a high number of mouse Cre lines available by using either the *Gfap*, *Glast*, *Blbp*, *Gli*, *Nes*, *Cx30*, *CX43 or S100B* promoters (for review see [134]). Genetics tools are also available to inducibly knockout gene expression with the CreERT2 system.

Additionally, researchers have used non-genetic methods to knockdown genes at the BBB. One promising technique is the delivery of siRNA into brain ECs. By high pressure tail-vein injection of a claudin 5 siRNA or by infecting a brain region stereotactically with a virus that produces a claudin 5 shRNA, Campbell *et al*. found that it was possible to knockdown *Cldn5* gene expression in brain ECs and consequently open the BBB to some extent [172-174]. They recently demonstrated that knocking down *Cldn5* at the mouse BBB leads to a decrease in cerebral edema after traumatic brain injury [175]. Other groups showed efficient delivery of exosome-associated siRNA [176] or nanoparticles coupled probes [177] to the CNS. Finally, it is also possible to use ultrashort pulsed laser [178] or ultrasound coupled with MRI to disturb the BBB and deliver molecules into the CNS [179].

## Potential tools for analysis of the BBB

As the boom of mouse genetic analysis continues, we can forecast that the generation of many novel mouse lines in the upcoming years will continue to advance our understanding of BBB function. Here we suggest a small number of tools that will aid in our understanding of BBB function during health and disease.

For BBB TJs, several interesting questions remain unanswered. There are multiple claudin family members expressed in CNS ECs, however it is unclear whether each claudin has unique functions within the TJ or whether each member provides an additive effect on limiting the permeability of the vessels. Developing knockout mice for each claudin expressed by CNS ECs, as well as double and triple *cldn* knockouts will allow us to address these questions. In addition, *cldn5* knockout mice die shortly after birth and thus it is not clear what the role of claudin 5 in the adult is or whether *cldn5* knockout mice die specifically due to lack of claudin 5 protein in CNS ECs or in other cells in the organism. Generation of a lox-p flanked *cldn5* allele would allow for temporal and spatial control of claudin 5 deletion to answer these questions. Furthermore, the knockout models are irreversible, and therefore generation of transgenic doxycycline-regulated claudin 5 shRNA would develop a model in which TJ protein expression could be reversibly modulated. Moreover, additional TJ proteins, such as the lipolysis-stimulated lipoprotein receptor (LSR) and tricellulin, that have recently been discovered in epithelial cells, have also been found to be enriched in CNS ECs compared to endothelial cells in non-neural tissues [26]. These TJs are expressed at the epithelial tricellular junction, where three cells meet [180,181]. It would be interesting to create the endothelial specific knockout of these TJs to understand their role in BBB formation and/or maintenance.

Our understanding of many BBB transporters has relied on the analysis of knockout mice in which the transporter is deleted from every cell. For instance *Abcb1a*/*Abcb1b*/*BCRP* triple knockout mice have been extensively utilized to study the role of these genes in xenobiotic protection. However, these genes are expressed in a variety of cell types in different tissues, and thus complete knockout of these transporters would globally change the localization of their substrates, and thus their specific function at the BBB may be obscured. Generation of mice with lox-p flanked alleles of different transporters would allow for analysis of their function specifically at the BBB.

In this review, we described several Cre lines that target ECs, including Tie2-Cre, VE-Cadherin-CreERT2, PDGFB-CreERT2, and others. Many of these *Cre* alleles target all ECs, and therefore it would be helpful to generate *Cre* alleles and *CreERT* alleles that specifically target CNS ECs. To do so, one could take advantage of the split-Cre system. In this system, the Cre is fragmented into two non-functional fragments and becomes active only when expressed in the same cells. Therefore, by using two different promoters, one can drive Cre expression (or CreERT2) in a very specific cell type [182,183]. For instance splitting the Cre to be driven by *VE*-*cadherin* and *Glut1* promoters would potentially allow for the specific targeting of CNS ECs. In addition, very little is known about the differences in the BBB at different segments of the vascular tree, from arteries to arterioles to capillaries to post-capillary venules to veins. Generating Cre, CreERT, and tTA lines that specifically target individual segments of the vascular tree would be of great use to the scientific community.

Several genetic tools have been utilized to eliminate pericytes by affecting PDGFBB/PDGFRβ signaling by generating knockout or hypomorphic alleles of *pdgfb* or *pdgfrb*. These knockout models affect pericytes during development, and thus it is not clear what the effect of acute pericyte loss in adults would be. Therefore, developing a genetic model to target pericytes in adults would allow us to further understand the role of pericyte-EC interactions. For instance, generating a *Pdgfrb*-rtTA allele coupled with a TRE-DTA allele would allow for doxycycline inducible ablation of Pdgfrb-positive cells. In this case doxycycline could be delivered systemically to target all PDGFRβ positive cells, or locally, for instance in the retina to specifically target retinal PDGFRβ positive pericytes, perhaps to mimic the loss of retinal pericytes during diabetic retinopathy. In addition, generating Cre/CreERT alleles that target specific subsets of pericytes would allow for the study of the relative contribution of each subtype of pericytes. Gortiz *et al*. identified that there were at least two types of pericytes, termed type A and type B pericytes [127], and were able to genetically mark type A pericytes with a GLAST-CreERT allele, however no such line has been developed to target type B pericytes. This might be more difficult as the molecular signature of different pericyte subtypes have not been described. The same can be said for astrocytes for which the complex heterogeneity among different subclasses of astrocytes is beginning to be understood. Developing Cre-lines that target specific functional subsets may be important for understanding the regional specificity of BBB regulation.

## Conclusions

The generation of mouse genetic models that target BBB structure and function have allowed us to gain a great deal of knowledge about this important physiological structure. These include models that target specific cells including ECs, pericytes and astrocytes, as well as identifying specific genes that are important for BBB formation and function. As the boom in mouse genetics continues we can expect the generation of many more genetic models that will continue to aid in the advancement of our understanding of the BBB.

## Abbreviations

ABC: ATP-binding cassette; AD: Alzheimer’s disease; Agt: Angiotensinogen; Ang: Angiotensin; ApoE: Apolipoprotein E; Aqp4: Aquaporin 4; BBB: Blood–brain Barrier; Bcrp: Breast cancer resistance protein; Btk: Bruton’s tyrosine kinase; CNS: Central nervous system; DTA: Diptheria toxin A; EAE: Experimental autoimmune encephalomyelitis; EC: Endothelial cell; Gal: Galactosidase; GFAP: Glial fibrillary acidic protein; GFP: Green fluorescent protein; HSV-TK: Herpes simplex virus thymidine kinase; MBP: Myelin basic protein; MCAO: Middle cerebral artery occlusion; MMP: Matrix metalloproteinase; MS: Multiple sclerosis; NPC: Neural precursors cell; PD: Parkinson’s disease; PIGF: Placental growth factor; Pgp: P-glycoprotein; rtTA: Reverse tetracycline transactivator; SHh: Sonic hedgehog; TJ: Tight junction; TRE: Tetracycline response element; tTA: Tetracycline transactivator; VEGF: Vascular endothelial cell growth factor; YFP: Yellow fluorescent protei

## Competing interests

The authors declare that they have no competing interests.

## Authors’ contribution

FS and RD wrote the manuscript. All authors have read and approved the final version of the manuscript.

## Supplementary Material

Additional file 1: Supplementary Table 1 Genetic mouse models to study the BBB. Click here for file
