# Supplementary material for: Genetic mouse models to study blood–brain barrier development and function
Source: Fluids Barriers CNS. 2013 Jan 10;10:3. doi: 10.1186/2045-8118-10-3 (PMC3675378; doi:10.1186/2045-8118-10-3)
Supplement: Additional file 1: Supplementary Table 1 — Genetic mouse models to study the BBB. [file 2045-8118-10-3-S1.pdf]

Supplementary Table 1. Genetic mouse models to study the BBB (references to be found in reference list in main text).

Genes	Targeted cell type	Studied age	BBB/CNS phenotype	Lethality	Reference(s)
Abcb1a	Full KO	Adult	Increased permeability to xenobiotics/drugs	None	[32]
Abcb1a/Abcb1b	Full KO	Adult	Increased permeability to xenobiotics/drugs	None	[35]
Abcb1a/Abcb1b/Bcrp	Full KO	Adult	Increased permeability to xenobiotics/drugs	None	[36] and Figure 3
Agt	Full KO	Embryo	Leaky BBB for serum proteins with less occludin at BBB.<br>Delay in repairing BBB after spinal cord injury	None	[113, 114]
ApoE	Full KO	Embryo	Leaky BBB in 2 week old and adult mice. Decrease in BBB TJs	None	[115, 116, 118]
Aqp4	Full KO	Embryo	BBB is normal.<br>Following MCAO, decreased cytotoxic cerebral edema and improved neurological outcome	None	[143, 144]
ca-β-catenin	Constitutively active in EC	Embryo	Precocious BBB maturation	None	[95]
loxp-β-catenin	Deletion in EC	Embryo	Required for angiogenesis into CNS and expression of GLUT1 at BBB	Embryonic	[94, 96, 99]
loxp-β-catenin	Deletion in EC	Postnatal	Required for sealing off BBB	None	[95]
Cldn1	Induced expression in EC	Adult	Seals BBB during EAE	None	[12]
Cldn5	Full KO	Embryo	Size selective leakiness of BBB	Perinatal	[9]
Dll1	Deletion in EC	Embryo	Loss of arterial identity during embryogenesis	None	[93]
Dll4	Full KO	Embryo	-/-, Severe vascular defects<br><br>+/-, Severe vascular defects	Embryonic<br><br>Embryonic,	[88, 89]<br><br>[88, 89]

				except ICR strain	
Dr6	Full KO	Embryo	Hemorrhages in forebrain, leaky BBB for sulfo-NHS-biotin, lower vascular density in hindbrain - Reduction of ZO1 at BBB	None	[101]
		Adult	Lower density of brain vasculature, leaky BBB for Evan's blue. Reduction of ZO1 at BBB	None	[101]
Dr6	Deletion in EC	Embryo	Hemorrhages in forebrain, lower vascular density in hindbrain - Reduction of ZO1 at BBB	None	[101]
Loxp-eGFP-stop-loxp-DTA	Expression in different subset of astrocytes	Embryo	Kill subset of ACs	Variable	[139]
E-selectin	Full KO	Adult	Doesn't affect the progression of EAE	N/A	[50]
Flt-1	Full KO	Embryo	Abnormal vessel development	Embryonic	[74]
Flk-1	Full KO	Embryo	Abnormal vessel development	Embryonic	[75]
Glut1	Full KO (promoter and exon 1)	Embryo	-/-, important morphological abnormalities<br><br>+/-, epilepsy and impaired motor activity	Embryonic<br><br>None	[41]
Glut1	Full KO (gene-trap)	Embryo	-/-, non studied	Embryonic	[42]
		Neonatal/ Adult	+/-, increase of MCT1 and MCT2 transporters in neonatal brain	None	[42]
Gpr124	Full KO or Deletion in EC	Embryo	Angiogenesis disruption in forebrain and ventral spinal cord with localized malformations and hemorrhages	Perinatal	[102-104]
GFP	Ectopic expression in EC	Embryo	Used for imaging	N/A	[26]

GFP	Ectopic expression in microglia	Embryo	Used for imaging	N/A	[147, 148, 150, 153]
GFP	Ectopic expression in MBP-reactive T-cells	Adult	Used for imaging	N/A	[154, 155]
H2b-GFP	Ectopic expression in arterial EC nuclei	Embryo	Used for imaging	N/A	[145, 146]
Icam1 ***(tm1Alb)***	Full KO	Adult	Attenuation of EAE symptoms	N/A	[55]
Icam1 ***(tm1Bay)***	Full KO (Deletion of exon 5)	Adult	Worsening of EAE symptoms	N/A	[55, 56]
Mmp2	Full KO	Adult	Attenuated CNS inflammation after stroke	N/A	[67]
Mmp9	Full KO	Adult	Attenuated CNS inflammation after stroke	N/A	[66-68]
Mmp12	Full KO	Adult	Attenuated CNS inflammation during TME	N/A	[69]
Notch1	Full KO	Embryo	Vascular defects	Perinatal	[82, 84, 85]
Notch4	Full KO	Adult	None	None	[83]
Notch1/Notch4	Full KO	Embryo	Vascular defects	Perinatal	[83-85]
Loxp-Notch1	Deletion in EC	Embryo	Angiogenesis defects	Embryonic	[82, 90]
caNotch4	Inducible, constitutively active in EC	Postnatal	Abnormal connections between veins/arteries	None	[27, 86]
		Embryo	Vessel enlargement in brain associated with hemorrhages and neurological damage	Embryonic	[27, 86]
Occludin	Full KO	Adult	Brain calcification	None (Infertile males)	[15]
Pdgfb	Full KO	Embryo	Absence of microvascular pericytes with capillaries microaneurysms	Perinatal	[123]
Loxp-stop-loxp-Pdgfb	Deletion of murine	Embryo	Attenuated coverage of pericytes. BBB leakiness	None	[119]

+<br>Human PDGFB	*Pdgfb* in EC with ectopic expression of human *PDGFB* in EC		for water, low- and high-molecular weight molecules due to a defect of transcytosis. Dysregulation of BBB-specific gene in EC and lost of AC endfeet polarization		
Pdgfrβ	Full KO	Embryo	Endothelial hyperplasia, abnormal EC structure and shape. BBB leakiness to biotin, upregulation of endothelial genes involved in vascular permeability or adhesion	Perinatal	[120, 122, 123]
Pdgfrβ hypomorphic alleles	Hypomorph in all cells	Embryo	Different number of pericytes. BBB leakiness linked to this number during development. In aged mice, BBB breakdown that led to neural degeneration	None	[120, 124, 125]
Pigf	Full KO	Adult	Increase BBB permeability to fibrinogen after hypoxia and delayed angiogenic response	None	[79]
P-selectin	Full KO	Adult	Decreased BBB breakdown during stroke. Doesn't affect the progression of EAE	N/A	[46]
PSGL-1	Full KO	Adult	Attenuated seizures in an epilepsy model. Doesn't affect the progression of EAE	N/A	[47, 49]
H-Ras, N-Ras, loxp-K-Ras	Full KO of *H-Ras* and *N-Ras* and inducible deletion of *K-Ras* in a subset of pericytes	Adult	Failed divison of Type A pericytes upon spinal cord injury	N/A	[127]
RFP	Ectopic expression in macrophages	Embryo	Used for imaging	N/A	[151-153]
Shh	Full KO	Embryo	Major abnormal anatomy throughout body including the CNS. Lower amount of TJ at the BBB in E13.5 embryos	Embryonic	[109, 111]

Shh	Ectopic expression in the dorsal neural tube	Embryo	CNS hypervascularization	None	[110]
Smo	Deletion in EC	Embryo	Leakage of BBB for serum proteins at E14 and P19. Adult BBB is permeable to exogenous dyes. Reduction of BBB TJs	None	[111]
Loxp-Smad4	Deletion in EC	Embryo	Deficit pericytes coverage, intracranial hemorrhage, BBB breakdown	Perinatal	[128]
HSV-TK	Inducible ablation of astrocytes	Neonatal	Ataxia, neuronal excitotoxicity, disorganization of Purkinje cells and radial glia	Inducible	[135]
Troy	Full KO	Adult	Mild leakage for Evan's blue	None	[101]
Vegf-A	Full KO	Embryo	Abnormal vessel development	Embryonic	[72, 73]
Loxp-Vegf-A	Deletion in NPC	Embryo	Lox/+, altered brain and retinal vascular development with a higher number of microglia/macrophage on the developing veins. Smaller size of cortical brain	None	[76, 77]
Wnt7a/Wnt7b	Full KO	Embryo	CNS angiogenesis deficits, vascular malformations and hemorrhage	Embryonic	[94, 96]
Wnt7a/loxp-Wnt7b	*Wnt7b* deletion in NPC and Full KO for *Wnt7a*	Embryo	CNS specific hemorrhages	Embryonic	[96]
YFP	Ectopic expression in a subset of neurons	Embryo	Used for imaging	N/A	[147, 149]
